# Comparison of influenza surveillance data from the Republic of Korea, selected northern hemisphere countries and  Hong Kong Special Administrative Region SAR (China) from 2012 to 2017

**DOI:** 10.5365/wpsar.2019.10.2.015

**Published:** 2020-09-30

**Authors:** Bryan Inho Kim, Ok Park, Sangwon Lee

**Affiliations:** a Division of Risk Assessment and International Cooperation, Korea Centers for Disease Control and Prevention, Cheongju-si, Republic of Korea.; b Division of Strategic Planning for Emerging Infectious Diseases, Korea Centers for Disease Control and Prevention, Cheongju-si, Republic of Korea.

## Abstract

Influenza surveillance is conducted in many countries; it is one of the most important types of infectious disease surveillance due to the significant impact and burden of the influenza virus. The Republic of Korea has a temperate climate, and influenza activity usually peaks in the winter as in other temperate-climate countries in the northern hemisphere. This descriptive study compared the influenza surveillance data from the Korea Centers for Disease Control and Prevention with that from other countries and areas in the northern hemisphere, namely China, including Hong Kong Special Administrative Region SAR, Japan and the United States of America, to identify seasonal influenza patterns from 2012 to 2017. Data on influenza-like illnesses (ILIs) and laboratory surveillance were collected from various sources; visual comparisons were conducted on the onset, duration and the peak timing of each influenza season based on subtypes. Correlation coefficients were estimated, and time differences for the beginning of influenza seasons between the Republic of Korea and other countries were measured. ILIs in North China and cases reported from Japan’s sentinel surveillance showed high correlations with the Republic of Korea. The number of confirmed influenza cases in Japan showed a high correlation with the laboratory-confirmed influenza cases in the Republic of Korea. We found that there are similarities in the influenza patterns of the Republic of Korea, Japan and North China. Monitoring these neighbouring countries’ data may be useful for understanding influenza patterns in the Republic of Korea. Continuous monitoring and comparison of influenza surveillance data with neighbouring countries is recommended to enhance preparedness against influenza.

The influenza virus is a respiratory pathogen that is transmitted through respiratory droplets. (*1*) During seasonal influenza epidemics, high attack rates cause a significant public health burden. (*2*) The infection is usually self-limited in young adults but can lead to severe infections in people in high-risk groups, including elderly people (> 65 years old), pregnant women, children aged 6–59 months and adults with chronic illnesses. (*3*)

The Republic of Korea is located in a temperate region where a seasonal pattern of influenza is normally observed. (*4*) The annual peak is usually in January. Since the establishment of the Republic of Korea’s influenza surveillance system in 2000, (*5*) the early prediction of seasonal influenza epidemics has been a major priority. The surveillance systems in China, including Hong Kong Special Administrative Region SAR, Japan and the  United States of America (USA) differ, but their overall structure and scope are similar. The influenza surveillance systems for all four operate year-round to detect influenza; however, their data have not been systematically compared and similarities and differences in patterns have not been identified. For this reason, this study compared the Korea Centers for Disease Control and Prevention (KCDC) influenza surveillance data with influenza surveillance data in other northern hemisphere countries.

## Materials and methods

### Study design

A descriptive study compared the Republic of Korea’s influenza surveillance data with that from China,  including Hong Kong Special Administrative Region SAR, Japan and USA from week 36 of 2012 to week 12 of 2017 (238 weeks total) to understand the relative onset, duration and peaks of seasonal influenza. China, including Hong Kong Special Administrative Region SAR, and Japan were selected because of their geographical proximity to the Republic of Korea. The USA was selected because it is located in the northern hemisphere, and it has a comprehensive influenza surveillance system. The selected countries and areas operate influenza surveillance year-round and have both an influenza surveillance system that monitors clinical symptoms such as influenza-like illness (ILI) and a laboratory-based influenza surveillance system. The KCDC’s definition of ILI is temperature (*3*)38 °C with cough or sore throat. There were some differences in ILI case definitions. China defines ILI as temperature (*3*)38 °C, either cough or sore throat and no laboratory confirmation of alternative diagnosis; (*6*) Hong Kong Special Administrative Region SAR (China) defines ILI as temperature (*3*)38 °C plus two of the following: sore throat, cough, rhinorrhoea, myalgia, arthralgia; (*7*) and the USA defines ILI as temperature (*3*)37.8 °C and cough and/or sore throat and without a known non-influenza cause. Japan’s ILI case definition is sudden onset of illness, temperature > 38 °C, upper respiratory inflammation systemic symptoms such as general fatigue or one of these clinical criteria and a positive rapid laboratory diagnostic test for influenza. (*8*) The ILI case definitions of each participating country were used in this study to determine seasonal influenza epidemics. (*9*) There were inherent differences in all of the influenza surveillance systems. For laboratory surveillance systems, per cent positive was used in all countries except Japan where the number of confirmed cases was used instead. In-depth statistical analysis was limited due to differences in surveillance system settings.

The surveillance system in the Republic of Korea is composed of 200 sentinel sites that report ILI cases and rates. All influenza data are reported on a weekly basis. The ILI rate in the Republic of Korea is defined as the number of ILI cases divided by the number of 1000 outpatients per week. Thirty-six sentinel sites also participate in the laboratory surveillance, sending respiratory specimens for confirmation and subtyping of influenza virus. In China, ILI consultation rates reflect the percentage of hospital visits attributed to ILI. In Hong Kong Special Administrative Region SAR (China), ILI rates are reported as cases per 1000 consultations in general outpatient clinics. In the USA, the rate of ILI is the national percentage of ILI patient visits to health-care providers. In Japan, the number of cases per sentinel site is reported.

### Data collection

Data were retrospectively collected through national weekly surveillance reports of each country or region, official web sites and the World Health Organization’s FluNet. (*10*-*15*) China produces two separate sets of surveillance data: one each for North and South China (not including Hong Kong Special Administrative Region SAR [China]), (*12*) and data from both sets were collected for the analysis.

### Data analysis

**Figure 1:**
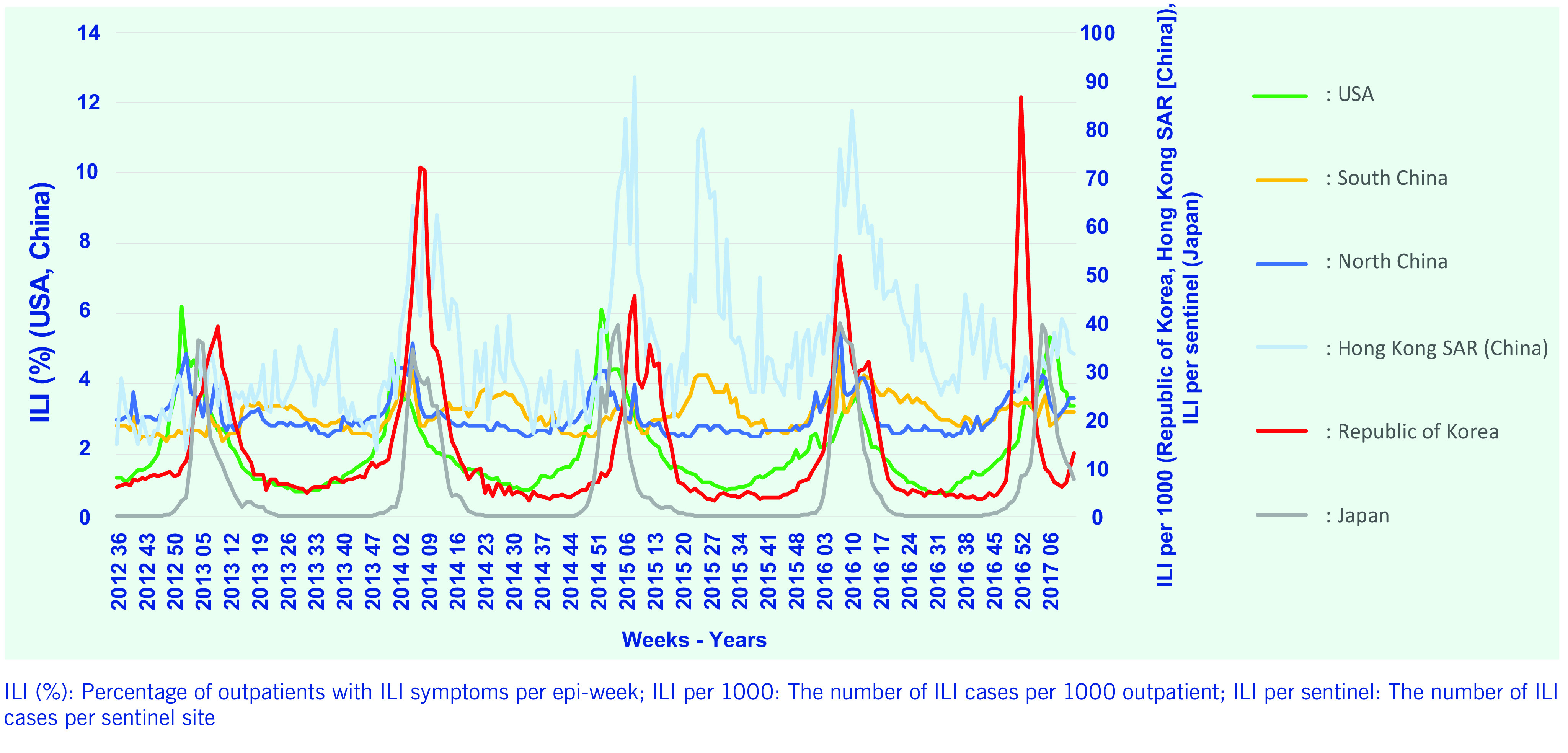
**ILI surveillance data, 2012–2017**

**Figure 2:**
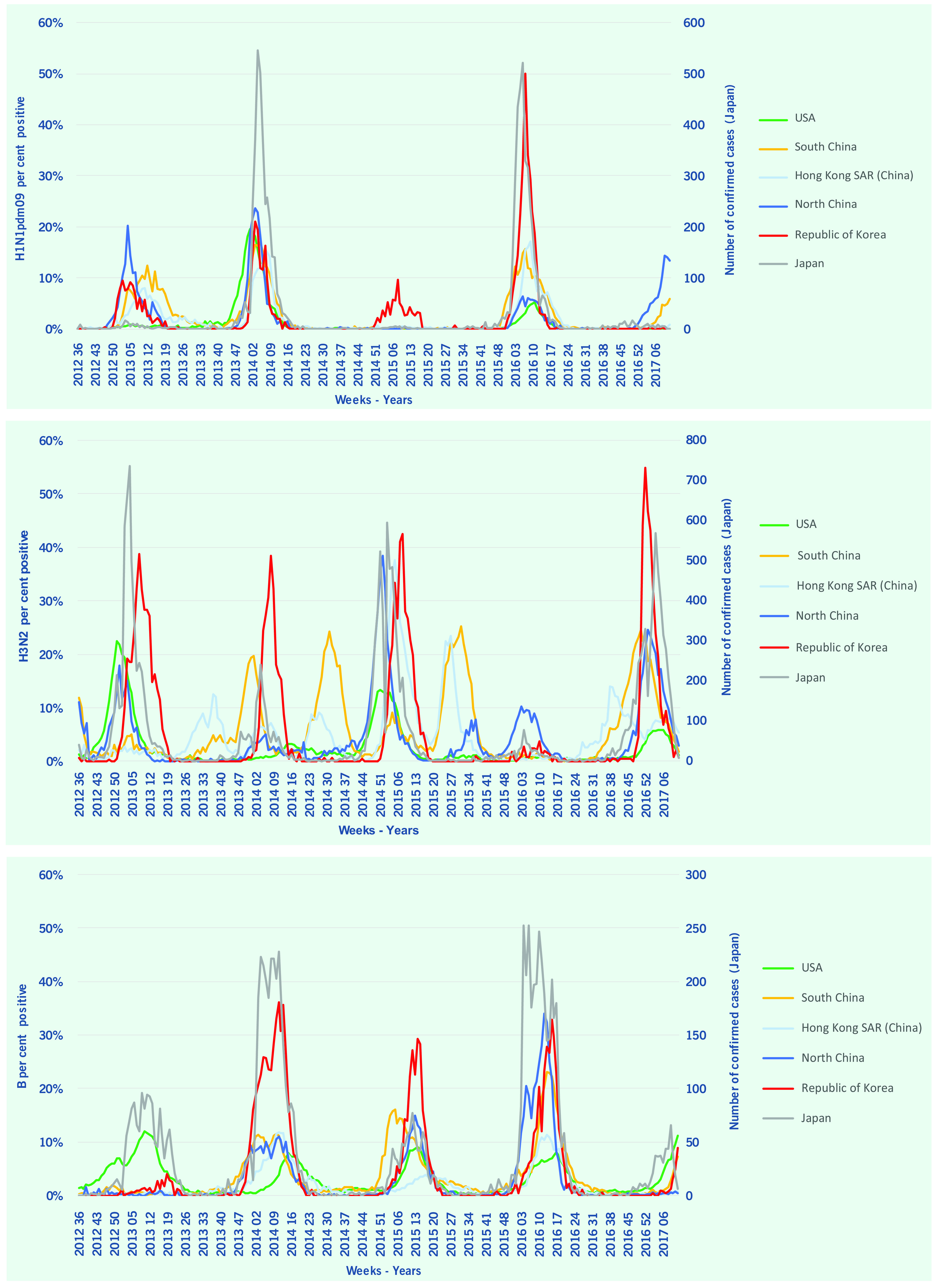
**Influenza laboratory surveillance data by virus subtype, 2021–2017**

**Figure 3:**
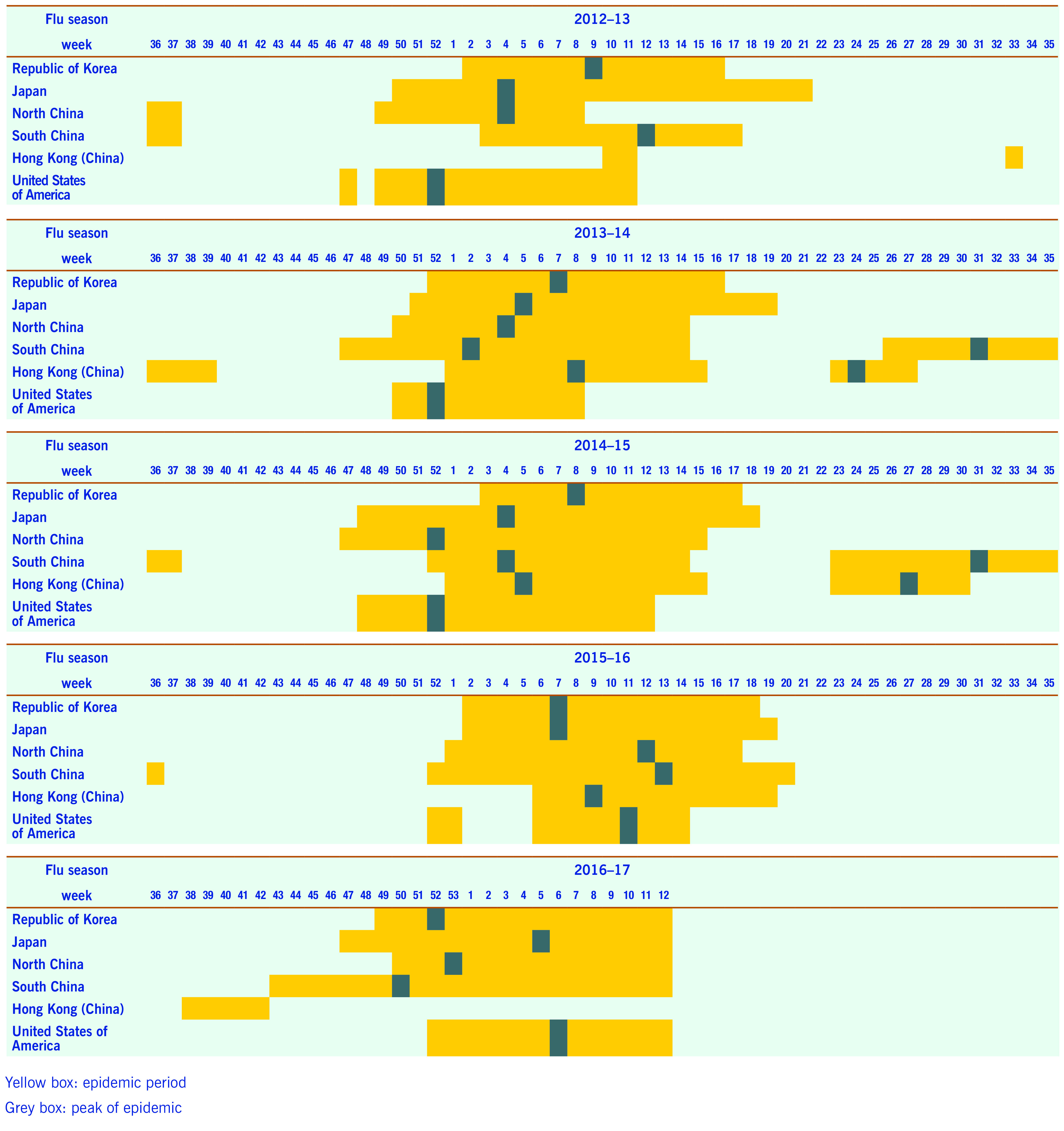
**Comparison of influenza seasons by country during the study period, 2012–2017**

Descriptive statistics including means, standard deviations, minimum and maximum values of ILI and per cent positive of influenza virus were calculated. Weekly surveillance data were plotted using the same epi-weeks to enable visual comparisons (**Fig. 1** and **2**). Onset, peak and the duration of each seasonal influenza epidemic were graphically presented by country for further comparisons (**Fig. 3**).

The week of onset was defined as the first week that exceeded the pre-defined level for countries using their own thresholds. The peak of the influenza season refers to the week that shows the highest ILIs (or cases per sentinel surveillance site for Japan) during epidemic periods of each influenza season.

As China and Hong Kong Special Administrative Region SAR (China) do not use an influenza epidemic threshold, the period in which influenza positivity rate was greater than 10% was used to define the epidemic period; this is normally used as the reference value of seasonal influenza in the Republic of Korea, the USA and other countries. (*16*, *17*)

Pearson correlation coefficients were calculated to compare the Republic of Korea’s surveillance data with the surveillance data of other countries and areas. We used weekly time lags (i.e. 1 week prior, 2 weeks prior, 3 weeks prior, 4 weeks prior) and considered typical influenza transmission patterns to find the best data sources. *P*-values less than 0.05 were considered statistically significant.

## Results

### ILI surveillance data

The mean weekly ILI rates varied by country during the study period. The mean rate for the Republic of Korea was 13.8 per 1000 outpatients (standard deviation [SD] 14.2); the mean rate for North China was 2.9% of ILI cases (SD 0.6%); the mean rate for South China was 3.0% of ILI cases (SD 0.5%); the mean rate for the USA was 1.9% (SD 1.2%); the mean rate for Hong Kong Special Administrative Region SAR (China) was 4.8 per 1000 consultations (SD 2.0); and the mean number of cases reported per sentinel site in Japan was 6.0 (SD 10.4). North and South China ILI rates had small variations (North: 2.3–5.6, South: 2.2–4.5) by year compared to the Republic of Korea and Japan.

The maximum per cent positive of influenza virus in the Republic of Korea was 71.7%; it was significantly higher than that of the other countries, which was around 40%. Among three influenza virus subtypes, the annual per cent positive of H3N2 was generally higher than the other two subtypes, except in the USA. In the USA, H3N2 and H1N1pdm09 showed similar proportions of positivity during the peak week (22.5% and 19.6%, respectively). No influenza viruses were detected during the intra-epidemic period (period between one influenza season and the next influenza season) in the Republic of Korea, North China or Japan (**Table 1**). In contrast, influenza was detected throughout the intra-epidemic perior in the USA.

**Table 1:** Descriptive statistics of the influenza surveillance data of the Republic of Korea, Japan, North and South China, Hong Kong Special Administrative Region SAR (China) and USA, 2012–2017

Country and data source	Unit	Mean	SD	Min	Max
Republic of Korea					
ILI	Per 1 000 outpatients	13.8	14.2	3.3	86.6
Influenza per cent positive (total)	% *	12.2	18.2	0.0	71.7
H1N1pdm09	% *	2.4	6.1	0.0	50.0
H3N2	% *	5.5	10.9	0.0	54.8
B	% *	4.3	8.5	0.0	36.2

Japan					
ILI	Cases per sentinel surveillance site	6.0	10.4	0.0	40.0
Influenza per cent positive (total)	Number of confirmed cases	144.8	204.2	0.0	1009.0
H1N1pdm09	-	31.7	92.3	0.0	546.0
H3N2		75.1	135.8	0.0	736.0
B		37.9	61.5	0.0	252.0

North China					
ILI	% **	2.9	0.6	2.3	5.6
Influenza per cent positive (total)	% *	9.4	10.7	0.0	42.1
H1N1pdm09	% *	2.6	4.1	0.0	23.7
H3N2	% *	4.3	6.4	0.0	38.5
B	% *	2.9	5.8	0.0	34.0

South China					
ILI	% **	3.0	0.5	2.2	4.6
Influenza per cent positive (total)	-	12.2	9.1	1.5	42.6
H1N1pdm09	-	2.4	6.1	0.0	17.0
H3N2	-	5.8	6.7	0.0	25.2
B	% *	3.6	4.8	0.0	23.0

Hong Kong Special Administrative Region SAR (China)					
ILI	Per 1 000 consultations	4.8	2.0	1.9	12.7
Influenza per cent positive (total)	-	8.9	7.8	0.3	38.7
H1N1pdm09	% *	1.9	3.5	0.0	17.1
H3N2	% *	4.9	6.2	0.1	37.5
B	% *	1.9	2.8	0.0	11.8

USA ILI					
**ILI**	% ***	**1.9**	**1.2**	**0.7**	**6.1**
**Influenza (total)**	**-**	**10.4**	**9.3**	**1.2**	**38.2**
A(total)	% *	7.5	8.3	0.3	31.4
H1N1pdm09	% *	1.2	3.3	0.0	19.6
H3N2	% *	2.6	4.1	0.1	22.5
B	% *	3.0	3.0	0.1	12.0

ILI = influenza-like illness, SD = standard deviation, Min = minimum value, Max = maximum value

* Percentage of influenza-positive samples over all tested clinical samples

** Percentage of hospital visits attributed to influenza-like illness

*** National percentage of patient visits to health-care providers for influenza-like illness

The ILI rates of countries and cases per sentinel surveillance site (Japan) data showed seasonality with winter season peaks during the study period (**Fig. 1**). The ILI rate in the Republic of Korea and cases per sentinel site in Japan showed sharp increases and clear peaks of seasonal influenza during the winter. In contrast, the USA’s ILI data showed gradual increases as well as decreases during influenza seasons every year. The USA’s ILI data showed earlier onset of epidemics four of the five previous influenza seasons. The Hong Kong Special Administrative Region SAR (China) and South China surveillance data demonstrated a pattern of summer epidemics.

[**insert** Figure 1]

### Laboratory surveillance data

Circulating subtypes varied among countries by each influenza season, and no clear patterns were identified. The per cent positive or the total number of confirmed cases (Japan) of H1N1pdm09 showed similar patterns among countries for onset and duration. The Republic of Korea was the only country that reported H1N1pdm09 during the 2014–15 season, but it was reported every year during the study period in the Republic of Korea. H1N1pdm09 showed a biennial pattern, being observed every other year in Japan and the USA. H3N2 showed more variations and irregularities compared with other subtypes, and the timing varied among countries and areas. Hong Kong Special Administrative Region SAR (China) and South China showed H3N2 epidemics in the summer seasons, but the timing varied in each influenza season. Influenza B virus showed lower per cent positive (or confirmed cases in Japan) and the onset was relatively delayed compared to other subtypes in all countries and areas (**Fig. 2**).

[**insert** Figure 2]

### Overall seasonal influenza pattern

There were yearly seasonal influenza epidemics for all countries and areas during the study period. The Republic of Korea, North China, Japan and the USA showed relatively similar influenza epidemic periods; there were interseason epidemics during the summer period in South China and Hong Kong Special Administrative Region SAR (China) (**Fig. 1** and **3**).

Pearson correlation (r) analysis demonstrated that most of the data from other countries were significantly correlated with the Republic of Korea’s data (**Tables 2** and **3**). There was a relatively higher correlation of ILI in North China (r = 0.54, *P* < 0.0001) and Japan’s sentinel surveillance cases (r = 0.60, *P* < 0.0001) with the ILI of the Republic of Korea. The number of confirmed influenza cases in Japan showed a high correlation (r = 0.71, *P* < 0.0001) with the Republic of Korea’s laboratory surveillance data.

**Table 2:** Pearson correlation coefficient between the Republic of Korea ILI data and time lag surveillance  data of other countries/areas, 2012–2017

Time lag	North China	South China	Hong Kong Special Administrative Region SAR (China)	Japan^a^	USA
No time lag	0.54 (*P* < 0.0001)	0.15 (*P* 0.020)	0.38 (*P* < 0.0001)	0.60 (*P* < 0.0001)	0.40 (*P* < 0.0001)
1 week time lag	0.59 (*P* < 0.0001)	0.14 (*P* 0.030)	0.35 (*P* < 0.0001)	0.60 (*P* < 0.0001)	0.43 (*P* < 0.0001)
2 weeks time lag	0.63 (*P* < 0.0001)	0.15 (*P* 0.019)	0.34 (*P* < 0.0001)	0.59 (*P* < 0.0001)	0.47 (*P* < 0.0001)
3 weeks time lag	0.64 (*P* < 0.0001)	0.13 (*P* 0.041)	0.30 (*P* < 0.0001)	0.55 (*P* < 0.0001)	0.51 (*P* < 0.0001)
4 weeks time lag	0.63 (*P* < 0.0001)	0.09 (*P* 0.148)	0.26 (*P* < 0.0001)	0.49 (*P* < 0.0001)	0.55 (*P* < 0.0001)

^a^ Cases per sentinel site

**Table 3:** Pearson correlation coefficient between the Republic of Korea influenza laboratory surveillance data and influenza laboratory surveillance data of other countries/areas, 2012–2017

Influenza specimens by type/subtype	North China	South China	Hong Kong Special Administrative Region SAR (China)	Japan^a^	USA
Influenza per cent positive (total)	0.64 (*P* < 0.0001)	0.58 (*P* < 0.0001)	0.67 (*P* < 0.0001)	0.71 (*P* < 0.0001)	0.48 (*P* < 0.0001)
H1N1pdm09	0.50 (*P* < 0.0001)	0.71 (*P* < 0.0001)	0.78 (*P* < 0.0001)	0.79 (*P* < 0.0001)	0.42 (*P* < 0.0001)
H3N2	0.34 (*P* < 0.0001)	0.14 (*P* 0.028)	0.33 (*P* < 0.0001)	0.54 (*P* < 0.0001)	0.26 (*P* < 0.0001)
B	0.75 (*P* < 0.0001)	0.76 (*P* < 0.0001)	0.84 (*P* < 0.0001)	0.75 (*P* < 0.0001)	0.39 (*P* < 0.0001)

***Source:*** Korea Centers for Disease Control and Prevention. (*15*)

^a^ Number of subtypes

The onset of influenza epidemics in Japan usually preceded that in the Republic of Korea by an average of 2.8 weeks, except in the 2015–16 influenza season. The onset of the influenza epidemic season started between one week (2013/2014) and seven weeks (2014/2015) earlier in Japan compared to the Republic of Korea. The duration of the influenza season was longer in Japan (average 21.5 weeks) than in the Republic of Korea (average 15.3 weeks). North China also preceded the Republic of Korea for the onset of the influenza epidemic season by one to eight weeks except for the 2016/2017 season.

The periods between the onset and the peak were significantly shorter in the Republic of Korea compared to Japan; in the 2016/2017 season it took only four weeks to reach the peak. The 2016/2017 season, was unique as the Republic of Korea and Japan experienced earlier onsets of seasonal influenza than in other years. Summer epidemics in Hong Kong Special Administrative Region SAR (China) and South China occurred in 2013/2014 and 2014/2015. However, this pattern was not observed in the 2012/2013 or 2015/2016 seasons. The summer epidemic was delayed and eventually started in the beginning of 2016/2017 season in Hong Kong Special Administrative Region SAR (China). An early epidemic in South China was observed and it may have influenced the earlier beginning of seasonal influenza in the 2016/2017 season in the Republic of Korea and Japan. The USA usually reported earlier onsets compared to other countries, but the pattern reversed after the 2015/2016 season (**Fig. 3**).

[**insert** Figure 3]

## Discussion

The study results indicated that Japan and North China had similar trends and tended to have earlier influenza onsets than the Republic of Korea. These countries and areas are located in East Asia, and geographical proximity might have resulted in similar patterns of seasonal influenza in both countries. (*18*) Also, similarities in climate conditions of the countries might explain the similar influenza surveillance results. We also found that the influenza data in the Republic of Korea and Japan varied more than it did in other countries. In North China, clear peaks in the winter season were also observed, but there were smaller ranges of ILI rates (differences between maximum and minimum) compared to the Republic of Korea and Japan. Influenza was reported throughout the year in South China and Hong Kong Special Administrative Region SAR (China) based on laboratory surveillance data, presumably due to their geographic locations in lower latitudes and closer to the equator. (*19*) Among subtypes, influenza B and H1N1pdm09 showed better correlation than the H3N2 subtype. This may be related to the irregularities of the H3N2 subtype and relatively large variations.

Even though this study covered fewer than five influenza seasons, our findings suggest that there potentially may be similarities in epidemic patterns in Japan, North China and the Republic of Korea. It is noteworthy that the onset of seasonal influenza epidemics in Japan tends to precede the onset in the Republic of Korea. The influenza virus shows clear seasonal trends in countries with temperate climates, and the correlation analysis showed statistically significant results. Nevertheless, the high correlation of ILI and confirmed cases in Japan and the Republic of Korea and in North China and the Republic of Korea suggests that there are similarities in the influenza patterns of these countries and areas.

There are some limitations to this study. As Japan uses a unique case definition for influenza surveillance, direct comparison with other countries and areas is somewhat limited. Although the case definitions for influenza surveillance were generally similar for the other countries, each system operates within different settings, potentially contributing to differential sensitivity and specificity for detecting influenza cases. (*20*) Surveillance systems in each country may also have been updated during the study period. The direct comparison of these diverse data may not fully capture or sufficiently explain the differences in patterns among countries. Laboratory surveillance data are also more likely to be affected by variations in surveillance system settings as they are strongly associated with the number of specimens tested. Also, annual influenza vaccination coverages of each country were not taken into consideration in the analysis due to the lack of access to the vaccination data. Despite the inherent discrepancies and potential lack of representativeness due to sentinel surveillance systems, these were the best national influenza data available.

Given the results of this observational study, additional studies to evaluate and validate the potential relationships among countries or regions are needed. Further study for longer period of influenza seasons with additional countries is needed to achieve more generalized outcomes.

## Conclusions

We found that there are similarities in the influenza pattern of the Republic of Korea, Japan and North China. Monitoring influenza patterns in Japan and North China may be useful for understanding influenza patterns in the Republic of Korea. Monitoring and comparing influenza surveillance data with neighbouring countries needs to be continued both for better understanding of influenza patterns and for possible earlier detection of onsets of seasonal influenza.
